# P02.68. Usefulness of Chinese herbal medicine in advanced cancer outpatients: a study on efficacy, tolerability and quality of life

**DOI:** 10.1186/1472-6882-12-S1-P124

**Published:** 2012-06-12

**Authors:** Y Wong, S Lo, K Wong, W Chan, Z Liang, C Che

**Affiliations:** 1 School of Chinese Medicine, The Chinese University of Hong Kong, Hong Kong, Hong Kong; 2 Tuen Mun Hospital, Hong Kong, Hong Kong; 3 The Pok Oi Hospital,, Hong Kong, Hong Kong

## Purpose

This study is to assess the effects of Chinese herbal medicine on aspects of health-related quality of life in patients with heterogeneous advanced cancer.

## Methods

It was a single-armed, prospective, pre-post and open-label observational study. At the department of oncology in a public hospital, 47 patients who failed pervious conventional therapies were recruited to receive one consultation per week (±2 days) over a study period of eight weeks. Two quality of life instruments, EORTC QLQ-C30 and MOS SF-36, were used to assess HRQOL in patients who were interviewed face-to-face at baseline, on Day 29 and Day 57. Any adverse events were also reported to assess the safety of Chinese herbal medicine.

## Results

Thirty-two patients were finally available for data analysis of quality of life. Mean score of the global health status of QLQ-C30 increased from 54.95 ± 9.15 (out of a maximum 100 points) at baseline to 67.45 ± 7.21 points on Day 29, and then decreased to 62.50 ± 7.97 points on Day 57 (F= 5.81; p<0.05). Several measures in the questionnaires also demonstrated improvements over the whole treatment such as emotional function, insomnia and constipation, but without reaching statistical significance. There were no significant changes in complete blood count, liver function tests and renal function tests. Sixteen cases of serious adverse events were reported but none of them was suspected to have a causal relationship with the Chinese herbal medicine used in the study.

## Conclusion

After the two-month Chinese herbal medicine treatment, the quality of life and symptoms of advanced cancer patients showed an overall improvement. Chinese herbal medicine is potentially effective for improving quality of life of advanced cancer patients during the palliative period.

